# Impact of COVID-19 on Surgical Interventions and Medical Practices in Pediatric Otolaryngology: A Narrative Review

**DOI:** 10.7759/cureus.23835

**Published:** 2022-04-05

**Authors:** Abdullah Jamal, Maryam Safar, Mohammad Tarakmeh, Mohammad Jamal, Khaled Alsaadi, Ali Safar

**Affiliations:** 1 Department of Otolaryngology, Zain Hospital, Kuwait City, KWT; 2 Faculty of Dentistry, Health Sciences Center, Kuwait University, Kuwait City, KWT; 3 Department of Family Medicine, Ministry of Health, Kuwait City, KWT; 4 Al Bahar Eye Center, Ibn Sina Hospital, Kuwait City, KWT

**Keywords:** covid-19, surgical interventions, pediatric otolaryngology, medical practices, ear nose and throat

## Abstract

The novel coronavirus disease 2019 (COVID-19) pandemic has become a major public health challenge. All types of elective and semi-urgent medical care and procedures have been discontinued during the pandemic to maintain the capacity to care for patients with this disease. The pandemic has had a significant impact on almost every medical field, including pediatric otolaryngology. This review highlights the impact of COVID-19 on surgical interventions and medical practices in pediatric otolaryngology owing to its direct association with ear, nose, and throat disorders, with an emphasis on immediate and potential long-term transformations in clinical practice. We reviewed several articles and scientific websites and summarized the currently available evidence and best practices for safety in the field of otolaryngology during the COVID-19 pandemic. Extensively discussed issues in pediatric otolaryngology include surgical interventions, medical practices, modes of transmission of COVID-19, personal protective equipment, and duration of exposure. Otolaryngologists should preserve their integrative medical approaches and subspecialty expertise during the COVID-19 pandemic. There has been a marked change in the approach to managing pediatric ear, nose, and throat conditions, both in the outpatient department and operating room, during the COVID-19 pandemic. The pandemic requires a great deal of flexibility and necessitates exploring new opportunities to create a safe and patient-friendly environment for children with otolaryngology problems. Many of the precautions implemented will remain necessary until a robust evidence shows the pandemic has come to an end.

## Introduction and background

The first case of coronavirus disease 2019 (COVID-19) was reported in December 2019 in Wuhan, China. COVID-19 has a lower mortality rate than severe acute respiratory syndrome (SARS) and the Middle East respiratory syndrome (MERS). However, it has a higher prevalence and longer incubation period, and can be transmitted asymptomatically. Globally, it has resulted in a large number of deaths. The World Health Organization (WHO) declared COVID-19 outbreak a pandemic on March 11, 2020 [1].

Since procedures and investigations involving the upper aerodigestive tract have a high likelihood of generating aerosols, ear, nose, and throat (ENT) surgeons are at a high risk of transmission [2]. Many professionals have recommended that only emergency procedures be performed and that all appropriate personal protective equipment (PPE) be used by healthcare workers during the COVID-19 pandemic [3].

In the human aerodigestive tract, the presence of foreign bodies is among the most common ENT emergencies in children [4]. These emergencies can lead to fatal outcomes in the absence of timely intervention. Thus, dealing with airway emergencies in pediatric patients during the COVID-19 pandemic is challenging [5].

The pandemic has profoundly impacted healthcare and the entire medical community, including pediatric otolaryngology professionals. As a result of limited resources and social distancing measures, the daily practice of pediatric otolaryngology has changed abruptly, requiring rapid adaptation to protect the health of providers and the financial viability of their practices. The outcomes of these modifications included the development of telemedicine, several preventive protocols to limit exposure to potential aerosol-generating medical procedures (AGMPs), and a national discussion on how to resume “normal” practice after the pandemic [5].

This review aimed to highlight the impact of COVID-19 on surgical interventions and medical practices in pediatric otolaryngology, with a clear focus on the potential long-term transformations in practice.

## Review

COVID-19 symptoms and transmission

The rapid development of the pandemic produces new data about the coronavirus and its effects on nearly a daily basis. Although our understanding was initially derived from several anecdotal reports and empirical evaluations, it has evolved to rely on more robust scientific data. Many scientific publications have been released online, and scientific societies have published their recommendations on the practice of otolaryngology in the era of COVID-19 [6].

The common symptoms of COVID-19 include shortness of breath, cough, and fever, whereas sore throat, sputum production, and muscle pain are less common symptoms. While most SARS-CoV-2 infections follow a benign course with few symptoms [7], some cases progress to more severe forms of the disease, such as severe pneumonia and multiple organ dysfunction. People who develop acute respiratory distress syndrome may experience thromboses, septic shock, and multiple organ failure [8].

The virus usually spreads between people via close contact, often through respiratory droplets by means of talking, sneezing, or coughing. The infected respiratory droplets typically enter the body through the nose and mouth. The conjunctiva is another potential portal of entry. These droplets also fall onto surfaces or the ground, and, in rare cases, some people may become ill by touching contaminated surfaces and then touching their mucous membranes [9]. Attention should be given to all possible modes of transmission for healthcare workers, and appropriate protective and hygiene measures should be taken [10].

Otolaryngologists and paramedics are high-risk groups for SARS-CoV-2 infection due to their direct contact with infected patients. They are vulnerable to direct transmission of the virus through mucus and airborne particles during clinical examinations, surgeries, and other interventions in the head and neck area. Evidence from several countries, including China, Italy, Iran, and the United Kingdom, suggests that ENT physicians are at the highest risk of contracting severe acute respiratory syndrome coronavirus 2 (SARS-CoV-2) infection, particularly when proper PPE is not used. Unfortunately, many doctors, nurses, and other medical staff worldwide have fallen victim to and died because of this pandemic. Therefore, precautions are recommended for ENT physicians in various procedures during the COVID-19 pandemic [11].

COVID-19 in children

Based on reports from China, South Korea, Italy, and the United States, the WHO announced that the incidence of COVID-19 in patients < 19 years of age was lower than that in adults [12]. In a Chinese study of 44,672 cases, only 2% of patients were < 19 years old and 0.9% were < 10 years of age [13]. In a South Korean study, 4.8% of patients were aged < 19 years, while only 15.9% were < 9 years old [14]. In Italy, 1.2% of patients were < 18 years of age among 22,000 cases [15]. Moreover, in the United States, only 5% of 4,000 confirmed cases were children. Infection may occur at any age among children, and the average reported age is seven years [14].

Although children and adults develop similar symptoms when infected with SARS-CoV-2, the symptoms are usually mild in children and are similar to cold symptoms. Most babies recover within one to two weeks [1]. According to a nationwide case series of 2,135 pediatric patients with COVID-19 reported to the Chinese Center for Disease Control and Prevention, the prognosis of COVID-19 is very good in children as 90% of children have mild or no symptoms, 5.2% have hypoxia, and only 0.6% experience acute respiratory distress. Nevertheless, children aged < 5 years appear to be more likely to develop a severe form of the disease. In the same series, the prevalence of the most severe form of COVID-19 was 10.6% in children aged < 1 year [13]. The first reported case of a child aged < 1 year with COVID-19 was a 55-day-old infant [16].

Despite their milder symptoms, children may have a higher viral load in the upper and lower airways, thus necessitating specific precautions for pediatric ENT surgeons [2]. As in adults, infection in children occurs by direct person-to-person transmission via aerosols, droplets, hand contact, or contaminated surfaces.

During the pandemic, newborn hearing screening, diagnosis, and treatment must continue. Auditory brainstem response and/or other diagnostic audiological tests should be considered as primary care for patients. However, genetic testing, eye examinations, and other tests may be delayed unless they directly affect the patient's short-term management [10].

COVID-19 effects on surgical interventions and medical practices

COVID-19 and pediatric otolaryngology: The major transmission routes of COVID-19 are respiratory aerosols, droplets, or direct contact [17]; sneezing, coughing, talking, and even breathing can generate aerosols and droplets. However, there is debate about whether aerosol spread is an important form of disease transmission [18]. The WHO and Canadian public health agencies have recommended that healthcare providers take precautions against droplet/contact transmission and additional airborne-spread precautions when performing AGMPs [19].

Several medical procedures are likely to generate aerosols; however, the formation of aerosols and the burden of viable infectious particles within aerosols have not been well studied. A systematic review by Tran et al. examined the risk of transmission of AGMP-related acute respiratory infection in healthcare providers and found that endotracheal intubation and tracheostomy were associated with the transmission of SARS-CoV-1 in the 2003 outbreak, while nasogastric (NG) tube insertion, bronchoscopy, and body fluid aspiration were not. Due to their associations with viral transmission, endotracheal intubation and tracheostomy pose the highest risks [20].

Flexible nasopharyngoscopy/laryngoscopy (NPL) represents a challenging and confusing area in this field, as it is not reliably recognized as an AGMP. It was initially thought that nasopharyngoscopy only caused sneezing or coughing during the procedure [21]. Given that its invasiveness is similar to NG tube insertion, which was not found to pose a significant risk as an AGMP, it is sensible to extrapolate that flexible NPL is likely to be a low-risk procedure, even if aerosols are generated [20].

Likewise, there is no evidence that coughing or sneezing caused by nasopharyngeal sample collection increases the transmission risk of COVID-19. This procedure is also similar to flexible NPL and has the risk of causing coughing or sneezing; however, NPL is arguably less traumatic than a nasopharyngeal swab because it is performed under direct visualization [17].

Middle ear aspiration is a controversial procedure in this pandemic. Many studies have shown that viruses in the nasopharynx during upper respiratory tract infections can also be detected in middle ear fluids [22]. However, in the absence of evidence, the mastoid fluid and middle ear must be treated as though they contain SARS-CoV-2 in infected patients. There is evidence that open endotracheal aspiration, particularly in patients undergoing mechanical ventilation, may create aerosols [23]. In contrast, a recent cadaveric study demonstrated the absence of aerosol generation during nasal aspiration using 10F Frazier suction. Moreover, other studies have shown that suction has the potential to decrease aerosol diffusion during AGMPs [17].

Considering the risk of SARS-CoV-2 infection, only procedures that cannot be delayed for more than two months and for which there are no medical alternatives must be performed during the pandemic. In difficult cases (e.g., cholesteatoma and tonsil enlargement with severe obstructive sleep apnea syndrome [OSAS]), treatment should be discussed collectively, with a written choice recorded in the medical file. For patients managed at a private clinic, it is recommended to seek advice from the ethics committee of the appropriate national ENT professional board.

Reverse-transcription polymerase chain reaction (PCR) COVID-19 tests on nasopharyngeal swabs obtained within 48 hours before surgery demonstrate a 30‒40% false-negative rate [12]. Nevertheless, this test is useful and necessary if there are signs suggestive of a SARS-CoV-2 infection. Urgent procedures, such as those required for severe or progressive shortness of breath, bleeding, or severe infections, should not be delayed due to test outcomes.

The sensitivity of a chest computed tomography (CT) scan to detect SARS-CoV-2 infection is >90%. A chest CT scan should be performed routinely if a CT scan is required in the preoperative evaluation (e.g., for complicated sinusitis, mastoiditis, neoplasms, a cervical or pharyngeal abscess). Other cases should be discussed considering the patient’s age and symptoms and the availability of a CT scanner. In the event of a preoperative diagnosis of SARS-CoV-2 infection, all non-urgent surgeries must be postponed for at least 15 days, which is the quarantine period [12].

Impact on otolaryngology practice: As with all ENT surgeries during the pandemic, patients undergoing ear surgery must be tested for COVID-19 preoperatively. If a patient tests positive, the surgery must be suspended until the patient is cured of the disease [24]. In patients who have tested positive for COVID-19, the surgeon and the entire operating room (OR) personnel must take appropriate precautions. If drilling cannot be avoided, tips to reduce aerosols include reducing drilling, using a reduced irrigation volume, effective suctioning, and controlled hypotension to reduce bleeding. Moreover, various techniques have been utilized to cover the surgical field and perform drilling from behind a protective screen [25].

When surgery cannot be postponed in patients with COVID-19, the use of powered air-purifying respirators with appropriate PPE is recommended for all OR staff. The same approach applies to the surgical management of epistaxis in patients with COVID-19. The use of power tools, such as razors and drills, should be restricted or avoided as much as possible [26].

In patients with COVID-19, the use of a tracheostomy must be discussed by a multidisciplinary team and should be performed only when absolutely necessary [25]. Based on the available data, the advantages of early tracheostomy in patients with severe COVID-19 have not been elucidated [24]. Given the nature of COVID-19, tracheostomy in intensive care unit (ICU) patients with COVID-19 is not very important. This is, first, because interstitial pneumonia in COVID-19 is associated with a low amount of secretion, and, second, because COVID-19 rarely allows for effective long-term intubation [27]. The possibility of performing a tracheostomy can be considered in all patients with COVID-19 with a stable pulmonary condition. Nevertheless, this should not be performed within two to three weeks of intubation and should preferably be performed following a negative PCR test. However, it must be avoided during periods of strict dependence on artificial respiration or respiratory instability [24].

When planning a tracheostomy in a patient with COVID-19, there are numerous factors related to surgery that must be considered [6,28]:

1. Appropriate PPE should be used.

2. Surgery should be performed at the bedside in a negative-pressure room in an ICU. If it is performed in the OR, it should preferably be performed with negative pressure in special regions in the OR complex.

3. An appropriate tracheal puncture tube should be used; an unlined, cuffed tube is ideal.

4. Reduce the exposure time to volatile secretions during surgery. Ensure complete paralysis of the patient throughout the procedure to prevent coughing. Stop mechanical ventilation directly before tracheal incision and reduce the usage of suction. If aspiration is required, it should be performed in a closed system with a viral filter.

5. The tracheostomy plan should be communicated to all team members ahead of time.

6. Carefully dispose of waste and decontaminate equipment after the procedure.

After surgery, important considerations to keep in mind include the following [6]:

1. The endotracheal cuff should be kept inflated, and care must be taken to avoid leaks.

2. Tracheostomy tube changes must be delayed until the patient’s COVID-19 test is negative or until the viral load is as low as possible. Subsequent changes to the endotracheal tube should also be delayed.

3. Tracheal aspiration should be performed by a closed suction system with a viral filter. Circuit disconnection should generally be avoided.

4. A heat exchanger should be used during weaning to prevent the spread of the virus between patients [6].

Challenges in pediatric otolaryngology during the COVID-19 pandemic

Because ENT surgeons are at particular risk of transmission, hospitals and doctors have had to adjust their routine work regimes to implement preventive measures. Numerous reports and protocols from various centers around the world have aimed to share their knowledge and experience with different clinical scenarios. The views discussed below should be considered to represent a dynamic view of an unprecedented situation that is constantly changing and calls for continual updates to management strategies [29,30].

Many factors must be considered when adjusting regional and institutional clinical practice based on the current pandemic situation. In many children's hospitals, the capacity for taking in pediatric patients has been diminished to meet the growing demand for care for adult patients with COVID-19. Employees must wear appropriate PPE for all AGMPs, as outlined by the WHO guidelines [31]. Moreover, it is necessary to make adjustments to the rooms and equipment. Altogether, these measures significantly reduce the number of children that can be seen in an outpatient setting. Therefore, at the time of writing of this report, international reports agree that medical activities should be limited to only urgent and time-sensitive cases [12]. Thus, in an outpatient setting, careful patient selection is required. Children’s caregivers should be contacted on an individual basis and examined over phone or video call to limit exposure and determine whether it is necessary to screen the child in person or if the visit can be rescheduled. Only in the event of urgent clinical problems or when a physical examination is necessary (such as for a suspected acute infection) should patients come to the outpatient clinic. Some cases may be managed by a phone visit, for example, follow-up for allergic rhinitis. By contacting the caregivers, the doctor can encourage contact with the department for urgent complaints or an unclear diagnosis and explain that ENT surgeons will always be available for essential care [2].

Hearing screening in newborns is a preventive healthcare process that is as important as childhood vaccinations. Depending on the local situation, the authors believe that these services should continue despite the COVID-19 pandemic except for newborns from mothers with suspected or confirmed SARS-CoV-2 infection. Newborns should undergo hearing screening immediately after birth if the mother tests negative for COVID-19. This requires the reorganization of screening procedures in order to reduce the risk of infection. Newborns who fail their hearing screenings should be referred to tertiary care centers for a confirmatory hearing test. In Belgium, reference centers expressed a commitment to continuing care of newborns with permanent hearing loss to reduce delays in hearing rehabilitation [2].

Children with a sudden onset or progressive worsening of wheezing or signs of respiratory failure should be observed in the emergency department by staff in full PPE without delay. The attending physician should then estimate the severity of the condition based on the history and physical signs. Depending on the individual case and the severity of the respiratory condition, airway endoscopy should preferably be avoided in an outpatient or emergency department setting. It should be scheduled under general anesthesia in the OR, with a pediatric ICU bed available for appropriate postoperative monitoring [2].

Adenotonsillectomy is a non-urgent procedure indicated for recurrent infections and mild OSAS. For children with mild OSAS, medical treatment should be attempted for three to six months. However, for children with severe OSAS, tonsillectomy may be the only treatment option. The decision to proceed with adenotonsillectomy on a semi-urgent basis should be limited to cases with severe OSAS confirmed by polysomnography or in children for whom continuous positive airway pressure is inappropriate or incompatible. This should be discussed by a multidisciplinary team in consideration of the parents’ preferences. Adenotonsillectomy should be preferred over continuous positive airway pressure, which poses the risk of spreading the virus into the surrounding air [12]. The role of the disease in such cases is unclear. According to the French Otolaryngology Society guidelines, ENT disease can be reduced in adults as they have a high potential for clinical improvement after tonsillectomy [32]. Children with severe OSAS are at an increased risk of developing respiratory disease after a tonsillectomy. This may lead to a longer hospital stay or require closer monitoring in the postoperative period. In addition, OSAS patients who do not have preoperative symptoms may develop symptoms after general anesthesia. In the absence of data at the time of writing this review, no specific technique is preferred. However, it should be made clear that coagulation and pelletizing techniques, as well as radiofrequency techniques, may generate more aerosols than do cold sclerotherapy-only techniques [12].

The placement of ventilation tubes is a non-urgent procedure. When daily practice can be gradually resumed, children at risk, such as those with serous otitis media and unilateral deafness or those with developmental delays, should be prioritized when rescheduling procedures. Moreover, consideration should be given to seeing children who had been scheduled for a ventilation tube placement visit at the outpatient clinic before rescheduling, as glue ears may fade spontaneously over time (during the spring season or with less exposure due to school and daycare closures during the pandemic) [2].

Cochlear implants for children with acute-to-severe sensorineural hearing loss may be considered as time-sensitive care. Whenever possible, the delay in implantation should not exceed 12 weeks, and children should continue to receive the first implant before the age of one year whenever possible. Children who develop bacterial meningitis after bilateral cochlear implantation should be treated without delay. Children with retraction pockets and indications of suspected cholesteatoma should be discussed on a case-by-case basis, depending on the extent and risk of complications, such as a labyrinthine fistula, meningocele, or facial paralysis. Indications for intranasal surgery should be limited to life-threatening conditions, given the high risk of viral particle spread during the procedure. In children with severe or progressive worsening of wheezing and failure of extubation, airway endoscopy may be mandatory for diagnosis and additional management [2].

A multidisciplinary respiratory team should discuss the indication and timing for airway endoscopy in these scenarios. Guidance is available from different communities regarding optimal personal protection in the OR during airway procedures and in cases in which an infectious agent is suspected. For airway endoscopy, several societies have advised using transparent curtains to reduce aerosols in the OR [33].

In the current COVID-19 era, elective tracheostomy should be delayed. It should be limited to cases with no other therapeutic solution, considering the high risk of viral particle spread during the procedure, postoperative care, and subsequent endotracheal tube changes. Over time and as we enter the middle stage of this pandemic, it will become necessary to consider elective tracheostomy in children. This should be a multidisciplinary decision, and the child should undergo preoperative COVID-19 testing. In addition to surgery, postoperative care and management should also be considered [34].

## Conclusions

The COVID-19 pandemic presents an extraordinary challenge to the medical community. Every patient in whom COVID-19 cannot be confirmed must be treated as a positive case. The way we practice medicine during the COVID-19 pandemic must be based on scientific evidence. Medical care must be allocated wisely to treat the most severe cases while minimizing the risks of long-term consequences and ensuring the safety of patients, physicians, and healthcare workers. Otolaryngologists and supporting personnel are particularly at risk of contracting COVID-19. As with all healthcare workers, their protection is essential to avoid a collapse of the healthcare system. Since the start of the COVID-19 pandemic, there has been a marked change in our approach to managing pediatric ENT conditions, both in the outpatient department and in the OR. Many of the precautions implemented will still be necessary for the coming months until highly sensitive tests and/or vaccines become widely available.

The COVID-19 pandemic will have a profound impact on healthcare around the world. Although the full ramifications of this disease have yet to be realized, the specific recommendations will guide otolaryngologists in treating pediatric patients in the face of an unprecedented global health crisis. With the rapid development of knowledge about SARS-CoV-2 infection, it is important to remain informed of relevant developments and recommendations. There is a need for an educational infrastructure that adapts to an ever-changing world.
